# An assessment of the impact of herb-drug combinations used by cancer patients

**DOI:** 10.1186/s12906-016-1372-x

**Published:** 2016-10-18

**Authors:** Saud M. Alsanad, Rachel L. Howard, Elizabeth M. Williamson

**Affiliations:** 1 Pharmacology Department, College of Medicine, Al Imam Mohammad Ibn Saud Islamic University (IMSIU), Road, Al-Nada, Riyadh, 13317-4233 Saudi Arabia; 2 School of Pharmacy, University of Reading, Whitekights, Reading RG6 6AP UK

**Keywords:** Complementary medicines, Alternative medicines, Cancer, Herb-drug interactions, Herbal medicines, Dietary supplements, Conventional medicines

## Abstract

**Background:**

Herb/Dietary Supplements (HDS) are the most popular Complementary and Alternative Medicine (CAM) modality used by cancer patients and the only type which involves the ingestion of substances which may interfere with the efficacy and safety of conventional medicines. This study aimed to assess the level of use of HDS in cancer patients undergoing treatment in the UK, and their perceptions of their effects, using 127 case histories of patients who were taking HDS. Previous studies have evaluated the risks of interactions between HDS and conventional drugs on the basis on numbers of patient using HDSs, so our study aimed to further this exploration by examining the actual drug combinations taken by individual patients and their potential safety.

**Method:**

Three hundred seventy-five cancer patients attending oncology departments and centres of palliative care at the Oxford University Hospitals Trust (OUH), Duchess of Kent House, Sobell House, and Nettlebed Hospice participated in a self-administered questionnaire survey about their HDS use with their prescribed medicines. The classification system of Stockley’s Herbal Medicine’s Interactions was adopted to assess the potential risk of herb-drug interactions for these patients.

**Results:**

127/375 (34 %; 95 % CI 29, 39) consumed HDS, amounting to 101 different products. Most combinations were assessed as ‘no interaction’, 22 combinations were categorised as ‘doubt about outcomes of use’, 6 combinations as ‘Potentially hazardous outcome’, one combination as an interaction with ‘Significant hazard’, and one combination as an interaction of “Life-threatening outcome”. Most patients did not report any adverse events.

**Conclusion:**

Most of the patients sampled were not exposed to any significant risk of harm from interactions with conventional medicines, but it is not possible as yet to conclude that risks in general are over-estimated. The incidence of HDS use was also less than anticipated, and significantly less than reported in other areas, illustrating the problems when extrapolating results from one region (the UK), in one setting (NHS oncology) in where patterns of supplement use may be very different to those elsewhere.

## Background

Many studies have recorded a high use of Herbal/Dietary Supplements (HDS) by cancer patients. The MD Anderson Cancer Centre, in USA, reported that 52 % of their cancer patients had used at least one form of Complementary and Alternative Medicine (CAM), and 77 % of those were using herbs and vitamins [37]. Between 25 and 47 % of ethnic Chinese cancer patients living in North America relied on herbal preparations as part of their cancer treatment [6]. In the US, a survey showed that about 63 % of outpatient cancer patients used HDS [35]. In the UK, a systematic review of 11 papers investigating the use of herbal medicines by cancer patients found the prevalence of herbal medicines use varied from 3.1 to 21.8 % among adults and from 4.1 to 20 % in paediatric patients [11].

In the absence of good efficacy data for most HDS, and given their popularity with patients, the most urgent current concern is assuring their safety (WHO [32]). Some herbal medicines have been extensively studied and there is clinical evidence for both potential benefits and risks (e.g. Hermann and von Richter [13]). Many pre-clinical studies which have been carried out to evaluate the safety of HDS in combination with licensed or prescribed drugs are not supported by the clinical evidence when carefully assessed (e.g. [17]).

Very few studies have been conducted to evaluate the safety of herbal medicines and their combinations used as self-medication, or as recommended by herbal medicine practitioners (Heinrich et al. [12]). The potential for interactions between herbs and other medicines may be higher due to the large number of components in the herb, compared to the single active substance of conventional medicines [9]. In addition, a single herb or product may be used to treat several conditions, and different herbs may be used to treat the same illness, making interactions difficult to interpret accurately. There is another obstacle, which is the lack of detail in many reports: in one study, about 2000 combinations from 4 electronic databases were screened to assess the probability of interaction, and of 108 cases identified as potential interactions, 70 % did not provide adequate data for this to be achieved. Only 13 % of cases were identified as ‘well-documented’, but warfarin was the conventional medicine most commonly involved, and St John’s wort was the herb cited in most interactions [9].

Assessing the safety and efficacy of HDS use is further complicated because many studies have reported that cancer patients often do not tell their healthcare providers about their CAM use [2, 34]. Ernst [7] reported that only 25 % of cancer patients using HDS received advice from their doctors and that communication about HDS was virtually non-existent. Side effects and drug interactions were the most common concerns expressed by the researchers [14, 21, 23, 29] but to date, many documented potential interactions have been extrapolated from in vitro or animal studies, or have not been described in an appropriate context [24, 34].

There are certainly herb-drug combinations which may result in serious consequences in cancer patients. For example, the concurrent use of enzyme inducers such as St. John’s wort (*Hypericum perforatum*) can affect many drugs: St John’s wort has been shown to cause a 44 % reduction in the time of imatinib (an anti-cancer drug) remains at therapeutic blood levels [20]. The aim of this study was to therefore investigate real-life HDS use by cancer patients and their experiences of benefit or harm. An individual assessment for each patient was used to assess the risk posed by the drug regime and HDS consumption.

## Methods

### Questionnaire distribution

Self-administered questionnaire surveys comprising open-ended and closed-ended questions (see supplementary information) were posted to eligible participants being treated for cancer at the Oxford University Hospitals Trust (OUH) and centres of palliative care at the Sobell House, Duchess of Kent House, and Nettlebed Hospice, located in the Thames Valley Area, UK. Cancer patients were identified by oncology and palliative care consultants at each study centre based on the following inclusion criteria: aged above 18 years, able to speak English, currently receiving treatment for their diagnosed cancer, and able to understand and fill out the questionnaire. Patients were excluded from the study if consultants felt they were unable to participate on medical grounds. All responses were anonymous unless the patients chose to provide contact details in order to receive feedback.

The questionnaire asked patients to record all of the conventional medicines prescribed by their GP, oncologist or palliative care specialist and, if they also took HDS, we asked them to list all products they had taken at the same time as their usual drug regime. Patients were not asked to select from a prepared list in order to explore the variety of products used and to see which substances they actually considered to be HDS. We also asked about their perceived experiences from these HDS use in order to retrospectively identify any experiences of benefit or harm. data such as sources of HDS and advice, and if they had taken HDS before being diagnosed, were gathered for separate analysis.

Questionnaires were posted to eligible participants and completed questionnaires returned to the study team in reply-paid pre-addressed envelopes over the study period (August 2013 – January 2014). Response rates were maximised by encouraging patients to complete and return the questionnaire even if they took no HDS; and patients who were taking HDS were offered the opportunity to receive feedback on the safety of their use. Due to financial constraints a single mailing was used. Where patients indicated that they would like feedback on their use of HDS, they supplied contact details, and a review letter providing information about the safety of the combination they were using was sent. If any risks were identified patients would be advised to contact their oncology or palliative care team to discuss in more detail, in accordance with the Ethical approval protocol.

### Assessing potential interactions between HDS and conventional medicines

Combinations of HDS and conventional medicines reported by participants were assessed using the system of classification or the potential for interaction and severity as described in Stockley’s Herbal Medicine’s Interactions (SHMI) (2013). A literature search was also carried out in case very recent data had been published or if a supplement was not included. SHMI is a comprehensive, evidence-based reference for interactions between herbal medicines/dietary supplements and conventional medicines which is regularly updated on-line. It rates interactions in three different categories:

- Action: whether or not any action needs to be taken to address the interaction.
- Severity: the likely effect on a patient of an unmanaged interaction.
- Evidence: the weight of the available evidence.

On the basis of these, one of five symbols is used to describe the combination, ranging from; ‘no interaction’, ‘doubt about outcomes of use’, ‘potentially hazardous outcomes’, ‘significant hazard’ and to ‘life-threatening outcomes’. These symbols are defined as follows:

 No interaction: the interaction has not been conclusively demonstrated or is not considered to be clinically significant.
 Doubt about outcomes of use: there is doubt about the existence or severity of a suspected interaction, but patients may need some guidance about possible adverse effects, and/or monitoring should be considered.
 Potentially hazardous outcome: there may be a potentially hazardous interaction, but the data is poor or sparse and conclusions are difficult to draw.
 Significant hazard: current use may result in significant hazard to patients and so careful monitoring or dosage adjustment is needed.

 Life-threatening outcome: the interaction is life-threatening, and concurrent use should be avoided.

If SHMI and a literature review raised no concerns, in that no reports of interactions between the drugs, and in the case of the herb, a related herb containing similar constituents, were cited, the combination was assessed as ‘no interaction’. If the combination showed evidence of similar pharmacological activity, for example if the drug and the supplement were known to both possess anti-hypertensive activity, then the combination was categorised as ‘doubt about outcomes of use’. Assessments of interactions between HDS were made, as well as between supplements and conventional medicines, as these had not previously been carried out.

### Data analysis

A respondent was defined as a user of HDS if they reported that they had used herbal medicines or dietary supplements. Responses were categorised according to patient sociodemographic, cancer diagnosis, previous use of HDS, specific HDS being taken, prescribed medicines taken concurrently, and the individual HDI assessments. The data obtained from the questionnaires were entered into the (SPSS V.21) to calculate summary statistics, for the proportions of participants in each category, with 95 % confidence intervals. All data entry was double checked. HDS use, conventional medicine use, and HDS-conventional combinations presented as median and the interquartile range (IQR), for each of these measures.

## Results

Three hundred seventy-five out of 1457 (26 %; 95 % CI 24, 28) cancer patients who were approached, having been identified by oncology and palliative care consultants at each study centre, participated in the study. Of these responders, 127 out of 375 (33.9 %; 95 % CI: 29, 38) reported using HDS, before or after their diagnosis with cancer. The other 235 responders did not use HDS, but gave details of their demographics, including their diagnoses. In our sample, females (74; 58.3 %) used HDS slightly more than males (53; 41.7 %); however, the responders were also predominantly female. Patients aged 60 years and above (74; 58.3 %) used HDS more than other age groups. Breast, prostate, and melanoma cancer patients were the most commonly HDS users (40; 31.5 %), (26; 20.5 %), and (22; 17.3 %) respectively. The demographic characteristics of participants, both HDS users and non-users, are shown in Table 1.

**Table 1:** Demographic characteristics and CAM practices used by patients

Characteristic	HDS users (out of 127) (%; 95 % CI)	Non-HDS users (out of 235) (%; 95 % CI)
Age		
20–30	2 (1.6; 0.4, 5.5)	0
31–40	6 (4.7; 2.2, 9.9)	8 (3.4; 1.7, 6.6)
41–50	14 (11.0; 6.7, 17.7)	27 (11.5; 8.0, 16.2)
51–60	31 (24.4; 17.8, 32.6)	44 (18.7; 14.3, 24.2)
61–70	50 (39.4; 31.3, 48.1)	77 (32.8; 27.1, 39.0)
> 70	24 (18.9; 13.0, 26.6)	79 (33.6; 27.8, 39.8)
Gender		
Male	53 (41.7; 33.5, 50.4)	132 (56.1; 49.8, 62.4)
Female	74 (58.3; 49.6, 66.5)	103 (43.9; 37.6, 50.2)
Cancer diagnosis		
Breast cancer	40 (31.5; 24.1, 40.0)	51 (21.7; 16.9, 27.4)
Prostate cancer	26 (20.5; 14.4, 28.3)	35 (14.9; 10.9, 20.0)
Melanoma	22 (17.3; 11.7, 24.8)	55 (23.4; 18.4, 29.2)
Renal cancer	4 (3.2; 1.2, 7.8)	18 (7.6; 4.9, 11.8)
Brain cancer	8 (6.3; 3.2, 11.9)	5 (2.1; 0.9, 4.9)
Gastro-oesophageal cancer	3 (2.4; 0.8, 6.7)	12 (5.1; 2.9, 8.7)
Pancreatic cancer	3 (2.4; 0.8, 6.7)	10 (4.3; 2.3, 7.7)
Bowel cancer	6 (4.7; 2.2, 9.9)	11 (4.7; 2.6, 8.9)
Other^a^	15 (11.8; 6.9, 19.0)	30 (12.8; 9.1, 17.6)
Healthcare type		
Oncology	110 (86.6; 79.6, 91.5)	211 (89.8; 85.3, 93.0)
Palliative	17 (13.4; 8.5, 20.4)	24 (10.2; 6.9, 14.7)

^a^Cancer diagnosis grouped based on the frequency. Other cancer types include non-Hodgkins lymphoma, mesothelioma, adenocarcinoma, thymoma, bladder, bone, testicular, bile duct, stomach, throat, lung, liver, buccal, omentum, ampullary cancer, ovarian, bone marrow, tonsil, anal, tongue and neuroendocrine cancers

HDS users reported 1255 herb-drug combinations, for a median rate of 11.5 (IQR, 2.5–14) per user. Overall, 101 different HDS were used concurrently with other medicines, for a median 1.5 (IQR, 1–4) per user, with 167 different conventional drugs, for a median of 1 (IQR: 1–6) per user. Glucosamine, cod liver oil, vitamins, omega 3 fatty acids, green tea, garlic, selenium, and fish oil were the most common HDS taken; the numbers of patients reporting these and others are shown in Table 2. Cod liver oil, omega-3 fatty acids and fish oil are related products but there are some differences in their composition and the way they are marketed, and patients cited them by product name.

**Table 2:** Frequencies of specific Herbal and Dietary Supplements (HDS) cited by cancer patients

Supplement cited	No taking the named HDS	As % of total responders (127)
Glucosamine	28	22.1
Cod liver oil	26	20.5
Multivitamins	21	16.5
Vitamin C	17	13.4
Omega fatty acids; Vitamin D	15	11.8
Green tea	12	9.4
Fish oil, Garlic, Selenium	9	7.1
Chamomile; Co Enzyme Q10	8	6.3
Calcium	7	5.5
Zinc	6	4.7
Echinacea; Evening primrose; Peppermint; Turmeric	5	3.9
Cranberry; Flaxseed; Ginger; Vitamin B	4	3.1
Aloe Vera; ‘Herbal tea’ (not specified); Iron; Magnesium; Probiotics; Senna; Wheatgrass; Ginkgo biloba; St John’s wort	3	2.4
Astragalus; Blue/green algae; Bromelain; Chondroitin; Ispaghula; Kelp; Lycopene; Plant sterols; Saw palmetto; SuperGreens®; Vitamin B12; Vitamin E	2	1.6
Kalms; Lecithin; Milk thistle; Mistletoe; Starflower oil; Pycnogenol®; Soursop juice; Vegepa E-EPA®; Melatonin, Osteocare®; Brewer’s yeast; Menopace®; Grapeseed extract; Phyto power®, Quercetin; Shiitake extract; Digest plus®, L-glutathione; Sheep sorrel root; Tea-tree oil; Red clover; Pomegranate; Propolis; Wormwood; Liver flush®; Bach remedies®; Chlorella; BioCare® acidophilus; BioCare® antioxidants; Artichoke; Acai; Lutein; Sea buckthorn; Raspberry leaf; Rooibos tea; Blackcurrant; Ginseng; Manuka honey, Vanilla; Potter’s cough pastilles; POMI-T; Fennel, Devil’s Claw, Coconut; Krill oil, Indole-3-carbinol; Boswellia, Apricot kernels; Serenagen Sleep®; Carotenoid complex; Flavonoids; Cruciferous plus®; Formula IV®; Ocuvite®; ‘Chinese herbal tea’ (not specified).	1	0.8

We also examined the HDS taken by patients with specific cancer types, to see if any group showed a preference for a specific HDS, and as such might be at particular risk. Breast cancer patients reported taking the widest range of DHS, citing over 24 different types of product, as shown in Table 3, and the most popular HDS were similar to those for the overall cohort of responders, i.e. cod liver oil, vitamins, glucosamine, omega 3 fatty acids and green tea. Prostate cancer patients reported using 15 different HDS; the most popular again being cod liver oil, glucosamine, vitamins, green tea and omega 3 fatty acids, as shown in Table 4. Melanoma patients reported using 9 different HDS products, again conforming to the general pattern of the responders overall, shown in Table 5. Renal, brain, gastro-oesophageal, pancreatic and bowel cancer patients also reported using a variety of different HDS but there were insufficient numbers of each diagnosis to analyse separately.

**Table 3:** Frequencies of specific HDS use by cancer diagnosis. Specific HDS use by breast cancer (BC) patients and comparison with overall frequency of use

Supplement	No of BC patients reporting each HDS (% out of 40 BC patients; % use overall in 127 HDS users, from Table 1)
Cod liver oil	9 (22.5; 20.5)
Vitamin D	8 (20; 11.8)
Glucosamine	7 (17.5; 22.1)
Multivitamins	7 (17.5; 16.5)
Omega-3 fatty acids;	5 (12.5; 11.8)
Vitamin B	5 (12.5; 3.1)
Green tea	4 (10; 9.4)
Calcium	4 (10; 5.5)
Chamomile;	3 (7.5; 6.3)
Co-enzyme Q10;	3 (7.5; 6.3)
Fish oil;	3 (7.5; 7.1)
‘Herbal tea’ (not specified)	3 (7.5; 2.4)
Wheatgrass	3 (7.5; 2.4)
Aloe vera	2 (5; 2.4)
Bromelain	2 (5; 1.6)
Cranberry	2 (5; 3.1)
Evening primrose	2 (5; 3.9)
Garlic;	2 (5; 7.1)
Kelp	2 (5; 2.4)
Peppermint	2 (5; 3.9)
Selenium	2 (5; 7.1)
Turmeric	2 (5; 3.9)
Vitamin C	2 (5; 13.4)
Zinc	2 (5; 4.7)

**Table 4:** Frequencies of specific HDS use by cancer diagnosis. Specific HDS use by prostate cancer (PC) patients and comparison with overall frequency of use

Supplement	No of PC patients reporting each HDS (% out of 26 PC patients; % use overall in 127 HDS users, from Table 1)
Cod liver oil	7 (27; 20.5)
Glucosamine	6 (23; 22.1)
Multivitamins	6 (23; 16.5)
Vitamin C	5 (19.2; 13.4)
Green tea	4 (15; 9.4)
Omega-3 fatty acids	3 (11.5; 11.8)
Selenium	3 (11.5; 7.1)
Co-enzyme Q10	2 (7.7; 6.3)
Evening primrose oil	2 (7.7; 3.9)
Fish oil;	2 (7.7; 7.1)
Garlic;	2 (7.7; 7.1)
Lycopene	2 (7.7; 1.6)
Saw palmetto	2 (7.7; 1.6
Vitamin B	2 (7.7; 3.1
Zinc	2 (7.7; 4.7)

**Table 5:** Frequencies of specific HDS use by cancer diagnosis. HDS reported use by melanoma patients and comparison with overall frequency of use

Supplement	No of ML patients reporting each HDS (% out of 22 ML patients; % use overall in 127 HDS users from Table 1)
Glucosamine;	12 (54.5; 22.1)
Cod liver oil	10 (45.4; 20.5)
Vitamin D	7 (31; 11.8)
Multivitamins	6 (27.3; 16.5)
Green tea	4 (18.2; 16.5)
Omega-3 fatty acids	3 (13.6; 11.8)
Selenium	3 (13.6; 7.1)
Calcium	2 (9.1; 5.5)
Zinc	2 (9.1; 4.7)

### Interactions between HDS and conventional medicines

Most of the herb-drug combinations (1225; 97.6 %) were categorised as ‘no interaction’, 22 combinations were categorised as interactions with “doubt about outcomes of use”, 5 combinations as interactions with “Potentially hazardous outcome”, one combination as an interaction with “Significant hazard”, and one combination as an interaction with “Life-threatening outcome”, as shown in Table 6.

**Table 6:** Assessment of Herb/Dietary Supplements (HDS) -Conventional Medicine (CM) Interactions

HDS	CM [no of patients^a^]	Possible interactions based on previous reports or theoretical grounds and their assessment^b^
HDI Category: “doubt about outcomes of use”		
Cod liver oil	Aspirin [4] Clopidogrel [1] Warfarin [1] Simvastatin [3] Atorvastatin [2] Rosuvastatin [1] Heparin [1]	Both have antiplatelet properties, but the combination did not cause any problems in any of the 4 patients and no previous clinical reports are available. Both have antiplatelet properties, but the combination did not cause any problems and no previous clinical reports are available. Cod liver oil and warfarin both increase INR. This combination has not caused any problems in the patient, no clinical reports are available, and was assessed as unlikely to be harmful. Both have cholesterol-lowering properties. The combination did not cause problems in the patients. As for simvastatin. As for simvastatin Cod liver oil has antiplatelet effects and increase INR, and heparin is an anticoagulant, but the but the patient did not experience problems, possibly due to being monitored for heparin effects.
Glucosamine	Insulin [1] Doxorubicin [1] Epirubicin [1] Paracetamol [4]	Endogenous glucosamine is involved in glucose metabolism but studies suggest that it is unlikely to affect diabetic control in patients taking insulin. Glucosamine has produced a modest resistance to doxorubicin in colon and ovary cancer cells in vitro but the effect has not been confirmed in vivo, and was assessed as unlikely to be harmful. As for doxorubicin, and therefore assessed as unlikely to be harmful. Glucosamine sulphate may reduce the efficacy of paracetamol (2 previous reports), by increasing paracetamol sulfate conjugation, but the combination did not cause any problems in our 4 patients.
Omega 3	Dipyridamole [1] Aspirin [4] Simvastatin [1] Pravastatin [1]	Omega-3 oils and dipyridamole all have antiplatelet properties. However, this combination did not cause ADEs in the patient, and was assessed as unlikely to be harmful. Omega-3 oils and aspirin both have antiplatelet properties. However, it did not cause ADEs in the patient, no other clinical reports are available so it was assessed as unlikely to be harmful. Omega-3 oils and simvastatin have cholesterol-lowering properties. However, the combination did not cause ADEs in the patient. As above (for simvastatin) as omega-3 oils and pravastatin have cholesterol-lowering properties.
Fish oil	Simvastatin [2] Warfarin [1]	Fish oil and simvastatin both have cholesterol-lowering properties. This combination did not cause any problems in the two patients taking it. Fish oil and warfarin both increase INR, but the patient did not experience problems, possibly due to being carefully monitored for warfarin effects.
Garlic	Atorvastatin [1] Heparin [1]	Garlic and statins lower plasma cholesterol but the but the patient did not experience problems. Garlic has antiplatelet effects and increases INR, and heparin is an anticoagulant, but the patient did not experience problems, possibly due to being carefully monitored for heparin effects.
Senna	Paracetamol [3]	It has been suggested that senna may reduce the absorption of paracetamol based on weak experimental evidence but our 3 patients did not experience problems.
St John’s wort	Omeprazole [1]	St John’s wort may lower plasma concentrations of omeprazole but there are no clinical reports.
Eicosapentaenoic acid (EPA)	Dalteparin [1]	EPA has antiplatelet properties and may add to the effects of anticoagulants such as dalteparin. However, the patient has not experienced any adverse effects.
HDI Category: “Potentially hazardous outcome”		
St John’s wort	Amitriptyline [1]	St John’s wort may lower plasma concentrations of amitriptyline but no harmful clinical reports have been recorded and the patient did not experience any problems.
Green tea	Clopidogrel [1]	It has been suggested that green tea may have additive effects with antiplatelet drugs such as clopidogrel but no clinical reports are available and the combination was assessed as not harmful.
Garlic	Lisinopril [1]	A single report of garlic with lisinopril in 1996 suggested the combination lowered blood pressure more than expected, but the patient did not experience any problems and garlic is taken widely.
Ginkgo	Omeprazole [1] Risperidone [1]	A clinical study found that ginkgo modestly induced the metabolism of omeprazole but a later study concluded that it was not clinically relevant. The patient reported no harmful effects. Priapism was previously reported in a patient taking risperidone and ginkgo. Risperidone alone causes priapism (rarely) and our patient did not experience this.
HDI Category: “Significant hazard”		
St-John’s Wort	Amlodipine [1]	St-John’s Wort is an inducer of CYP3A4 so the combination may lower plasma concentrations of amlodipine, decreasing hypotensive effects. However, the patient was being checked regularly and reported no ADEs, so the combination was assessed in retrospect as not harmful.
HDI Category: “Life-threatening outcome”		
St-John’s Wort	Sertraline [1]	The combination of St John’s wort and sertraline may lead to serotonin syndrome (reported in 4 previous studies). This combination is contra-indicated but the patient was no longer taking it.

^a^This is the number of patients exposed to the particular combination. In some cases, patients were also taking other recorded combinations

^b^The potential interactions and previous reports are taken from Stockley’s Herbal Medicine Interactions, except where not included in that reference, in which case other sources are given

Eighty-seven (75.7 %) HDS users reported that they had experienced benefits, with 82 attributing these to HDS. Only 4 (3.4 %) patients experienced problems, 2 believing they were caused by HDS; however, no adverse drug events (ADE) were recorded. 28 (24.7 %) patients responded that they had not experienced either benefit or harm during their HDS usage. 8 (6.3 %) patients had reported that they used HDS but did not report their experiences about HDS usage.

The HDI assessments showed that 11 HDS were involved in some risk of interaction: cod liver oil, St John’s wort, omega-3 oils, glucosamine, senna, green tea, EPA (eicosapentaenoic acid), fish oil, garlic, ginkgo, and evening primrose oil. Of the conventional medicines, 20 drugs were involved: aspirin, dipyridamole, clopidogrel, warfarin, dalteparin, heparin, insulin, omeprazole, sertraline, amitriptyline, risperidone, epirubicin, doxorubicin, paracetamol, co-codamol, amlodipine, lisinopril, atorvastatin, simvastatin, and pravastatin.

### Interactions between different HDSs

All combinations of HDS were deemed safe overall in the circumstances in which they were used, and the few cases which may have given cause for serious concern had already been resolved (see Table 7), usually because the patient had stopped taking the particular supplement before completing the questionnaire. Some herbal medicines have intrinsic properties which may case HDIs in large doses: for example, stimulant laxatives such as senna alter intestinal transit and in large doses or over a period of time may affect the absorption of any herb or drug taken at the same time, but these are predictable effects and can be avoided. The other issue is that supplements containing similar constituents may be taken together, for example evening primrose oil and star flower oil, which are both sources of gamolenic acid; however, as this compound is relatively safe the combination is unlikely to be harmful.

**Table 7:** Potential risks associated with the use of some HDSs used by cancer patients

HDS or combination of concern [no of patients exposed to potential risk]	^a^Comments on previous reports and assessment of risk
Soursop (Graviola) [1]	Herbal products made from the leaves or bark of soursop are sold as alternative treatments for cancer with no clinical evidence but on the basis of experimental studies showing selective cytotoxicity in some cancer cell lines. Their use is associated with atypical Parkinson’s disease, due to the content of acetogenins (especially annonacin-1, a mitochondrial complex I inhibitor) and the neurotoxic alkaloids reticuline and N-methylcoculaurine [4]. These compounds are not present to any great extent in the juice, which the patient had been drinking, so this practice was assessed as not harmful.
Evening primrose oil and Star flower oil in combination [1]	Evening primrose and star flower oils are both sources of gamolenic acid; however, gamolenic acid is a relatively safe substance and the combination is unlikely to be harmful.
Senna [3]	Senna increases intestinal transit and large doses can reduce the absorption of a drug. However, the 3 patients were taking opioids (codeine 2; tramadol 1) which cause constipation, and thus needed senna to counteract their effects.
Fish oil/cod liver oil/EPA/omega-3 fatty acid with garlic in combination [5]	The combination of fish oils (and by inference, unsaturated fatty acids) with garlic has been reported to have increased lipid lowering effects, but these are thought to be generally beneficial.
Apricot kernels [1]	Apricot kernels are promoted as an alternative treatment for cancer with no evidence of efficacy. They contain a glycoside called amygdalin which is toxic in large quantities as it releases cyanide gas, and poisoning has been reported [26]. The patient had not experienced any ADEs and had stopped taking the product, so intervention was not deemed appropriate.

^a^Comments on previous reports are taken from Stockley’s Herbal Medicine Interactions, except where not included, in which case other references are given

## Discussion

In a recent systematic review, Alsanad et al. [3] sought to determine the proportion of cancer patients deemed to be at risk from HDIs from the actual combinations they were taking, but only 5 studies were found which explored this issue specifically [8, 18, 20, 31, 37]. HDS are the most popular form of CAM used by cancer patients and have the greatest potential to cause harm, not only by causing adverse effects or by inappropriate use, but also by interacting with conventional medicines. There is thus a need to identify individual cancer patients taking HDS who might be at risk from HDIs, rather than making a global risk assessment based on population studies.

Our HDS safety assessments examined the actual combinations taken by each patient. Several combinations which would have been assessed as harmful on theoretical grounds did not actually produced an adverse effect in practice. It should be emphasised that our HDI assessments were not an estimation of risk, as originally intended, since they were done after the patient had taken the combination, and no patient reported any ADE. Instead it is an exploration of the levels of use of specific HDS with prescribed and/or licensed medicines and the patients’ experiences of taking these combinations.

Certain combinations were retrospectively judged as not clinically significant, despite having been previously categorised in other resources as carrying some risk, for example ‘Doubt about outcomes of use’, ‘Potentially hazardous outcomes’, or ‘Significant hazard’ as detailed in the Table 2. Our assessments also recorded the incidence of patients taking combinations which have been previously been judged to be inadvisable, but which did not result in a harmful outcome. In these cases, categorised as ‘Potentially hazardous outcomes’, ‘Significant hazard’ and ‘Life-threatening outcomes’, the patient would have been advised NOT to take the combination if they had enquired beforehand. No ill effects were reported, but whether this is because the risk is indeed theoretical or because the patient was simply fortunate, cannot be determined in a study of this type.

Cod liver oil, St John’s wort, omega 3 oils, glucosamine, senna, green tea, EPA (eicosapentaenoic acid), fish oil, garlic, ginkgo, and evening primrose were the HDSs involved in the risk of interaction with the conventional medicines: aspirin, dipyridamole, clopidogrel, warfarin, dalteparin sodium, heparin, insulin, omeprazole, sertraline, amitriptyline, risperidone, epirubicin, doxorubicin, paracetamol, co-codamol, amlodipine, lisinopril, atorvastatin, simvastatin, and pravastatin. Many anti-cancer drugs are metabolised by cytochrome P450 (CYP) enzymes, and especially CYP3A4, [22]. The interaction occurs if the HDS induces or inhibits the metabolizing enzymes or affects the drug transporter of the drug [19], but these should return to normal levels once the HDS use ceases [20].

A number of HDSs, such as ginkgo biloba, dan shen, liquorice, ma huang, have been implicated in reports of interaction with conventional medicines, and the drugs involved (warfarin, protease inhibitors and some anti-cancer drugs) have well-documented interaction profiles with other drugs [9, 15, 33]. St John’s wort has been involved in many HDI reports with other drugs [34] including anticancer agents, and for example, its interaction with imatinib occurs due to both induction of the metabolic enzymes CYP3A4 and CYP2C19 and the transporter of imatinib, leading to a lowering of plasma levels [27]. Therefore, it should certainly be avoided in cancer patients as a precaution [15, 25]. On the other hand, cancer patients often use garlic as a supplement, and although it has been suggested that garlic may alter metabolism of anti-cancer drugs such as docetaxel, a recent study found no in vitro interaction [10]. A risk of gastrointestinal bleeding has also been associated with garlic use [1, 28]: again, garlic is consumed by many people as food, and no ADEs reports have been published to date, so is unlikely to cause harm.

Cancer patients may sometimes feel the need for a mild sedative or tranquilliser, and the herb valerian is a very popular ingredient in herbal sleeping and relaxation products. Valerian has been said to “interfere in an unwanted way with oncological cancer therapy” or “can diminish the efficacy of cancer therapeutics” (e.g. Sloan Kettering Center 2012, cited by [17]). Kelber et al. examined the pre-clinical studies and concluded that ‘available animal and human pharmacodynamic studies did not verify any interaction potential’ and ‘there is no specific evidence questioning their safety, also in cancer patients’ [17]. In our study, the incidence of HDS use was less than anticipated, and significantly less than reported in other areas, again illustrating further problems when extrapolating results from one region (the UK), and in one setting (NHS oncology), where patterns of supplement use may be very different to those elsewhere.

Cancer patients regard HDS highly, as shown by many studies, and also the volume of sales of these products (Chrystal et al. [5]). The HDS identified in our study were used by patients with a broad range of diagnoses, and none was used only by a those in a particular group. Certain HDS were more popular in certain groups, as shown in Tables 3, 4 and 5, but the sample size was insufficient to draw any firm conclusions on these. Thus it is necessary to deal with the issue of HDIs in a realistic and pragmatic manner, because over-reaction to potential dangers and inaccuracies in reporting may cause patients to ignore even good advice on this subject. Another important issue is how the lack of evidence of HDS safety and efficacy should be addressed, as well as how the current evidence should be interpreted. In other words, there is a strong demand for an appropriate and robust framework for evaluating HDS safety and efficacy at different levels of patients’ needs, which includes social, epidemiological, and clinical studies [36].

It is crucial to alert and educate patients about the possibility of HDIs but it would be counter-productive to scare them with unfounded negative speculation at a time when they are particularly vulnerable. Clinicians should feel able to discuss any treatments that might be helpful, as well as warning of possible dangers, since there is a lack of awareness of these [30], and given the wide use of HDS, they need to be better informed. ADEs and HDIs related to HDS can be reported in the same way as adverse reactions to conventional medicines, in the UK via the Yellow Card Scheme, but happens infrequently. Whether this is because ADEs involving HDS are unusual or because of under-reporting is not clear, but if improved, would go some way to increasing the accuracy of the risk assessment of these combinations [33].

## Conclusion

Our study revealed that more than 100 different HDS were being used to manage cancer and other health issues, and our assessment of the numerous combinations of these with conventional medicines and other HDS showed that the majority of patients were not at great risk of HDIs. Most previous studies have assessed the safety of HDS use theoretically and without exploring the actual products used by cancer patients. Much of the available drug interaction evidence is based on pre-clinical studies, which do not always translate well into clinical experience, and in many case reports, data regarding the identity, composition, purity and even the dose of the herb is incomplete [16]. This may give an overestimate of risk based on speculation. Our survey was retrospective and most of the patients surveyed had been taking the combination for some time without experiencing any adverse events, but the sample size was not sufficient to conclude that the risk fir some supplements has been inflated on this evidence. As it would be unethical to deliberately give patients any combinations of drugs (including herbs) which may cause harm, the only feasible method of assessing risk at present is to collect evidence retrospectively from patients who are using or have used HDSs. This will provide documented evidence upon which healthcare professionals and cancer patients can based their judgement as to whether taking a particular HDS is advisable or not.

## Abbreviations

ADE: Adverse drug event
CAM: Complementary and alternative medicine
CYP: Cytochrome P
EPA: Eicosapentaenoic acid
HDI: Herb-drug interaction
HDS: Herbal\dietary supplement
IQR: Interquartile range
OUH: Oxford University Hospital
RGG: Research Governance Group
SHMI: Stockley’s herbal medicine’s interactions
WHO: World Health Organisation
